# Effects of the COVID-19 pandemic on trauma-related emergency medical service calls: a retrospective cohort study

**DOI:** 10.1186/s12873-021-00495-3

**Published:** 2021-09-09

**Authors:** Michael Azbel, Mikko Heinänen, Mitja Lääperi, Markku Kuisma

**Affiliations:** 1 Prehospital Emergency Care Services, Lapland Central Hospital, P.O. Box 8041, FI-96101 Rovaniemi, Finland; 2 Department of Emergency Medicine and Services, Helsinki University and Helsinki University Hospital, P.O. Box 340, FI-00029 HUS Helsinki, Finland; 3 Trauma Unit and Helsinki Trauma Registry, Helsinki University Hospital, P.O. Box 266, FI-00029 HUS Helsinki, Finland; 4 Department of Orthopaedics and Traumatology, University of Helsinki and Helsinki University Hospital, Helsinki, Finland

**Keywords:** Emergency medical service, Prehospital trauma, COVID-19, Alcohol

## Abstract

**Background:**

The COVID-19 pandemic has had profound effects on the utilization of health care services, including Emergency Medical Services (EMS). Social distancing measures taken to prevent the spread of the disease have greatly affected the functioning of societies and reduced or halted many activities with a risk of injury. The aim of this study was to report the effects of lockdown measures on trauma-related EMS calls in the Finnish capital area.

**Methods:**

We conducted a retrospective cohort study of all EMS calls in the Helsinki University Hospital (HUH) catchment area between 1 January and 31 July 2020. Calls were identified from the HUH EMS database. Calls were grouped into pre-lockdown, lockdown, and post-lockdown periods according to the restrictions set by the Finnish government and compared to the mean number of calls for the corresponding periods in 2018 and 2019. Statistical comparisons were performed using Mann-Whitney U-test for weekly numbers and percentages.

**Results:**

During the study period there was a total of 70,705 EMS calls, of which 14,998 (21.2%) were related to trauma; 67,973 patients (median age 61.6 years; IQR 35.3–78.6) were met by EMS. There was no significant change in the weekly number of total or trauma-related EMS calls during the pre-lockdown period. During the lockdown period, the number of weekly total EMS calls was reduced by 12.2% (*p* = 0.001) and the number of trauma-related calls was reduced by 23.3% (*p* = 0.004). The weekly number of injured patients met by EMS while intoxicated with alcohol was reduced by 41.8% (*p* = 0.002). During the post-lockdown period, the number of total and trauma-related calls and the number of injured patients intoxicated by alcohol returned to previous years’ levels.

**Conclusions:**

The COVID-19 pandemic and social distancing measures reduced the number of trauma-related EMS calls. Lockdown measures had an especially significant effect on the number of injured patients intoxicated by alcohol met by the EMS.

**Trial registration:**

Not applicable.

## Background

The first wave of the COVID-19 pandemic had profound effects on societies and the utilization of health care and EMS worldwide. Regarding trauma, there are reports of a marked decrease in the number of EMS activations [1–3] and the number of patients transported from prehospital care to hospitals [4] during lockdown periods, with numbers returning towards pre-pandemic levels after relaxation of lockdown measures. Trauma-related emergency department (ED) visits have decreased along with trauma surgery activity [5–8]. These changes have been attributed to the temporary reduction in road traffic, sports, and work-related activities during lockdown.

Finland experienced a rapid increase in COVID-19 incidence during week 11, starting 9 March 2020. To retard the spread of the disease, the Finnish government enacted several nationwide measures. The resulting lockdown caused widespread and drastic changes in the functions of Finnish society and affected work, education, healthcare, and social life, among others [9].

As the total number of EMS missions decreased during lockdown, we hypothesized that there would have been a reduction in trauma-related missions, especially traffic-related, due to reduced commuting and traffic volumes [10]. The closure of bars and nightclubs might have also resulted in a reduction of alcohol-related violence and injuries, as under normal circumstances a significant percentage of patients transported by EMS or admitted to emergency rooms are intoxicated [11]. The reduced rates of ED visits might also reflect EMS non-conveyance rates, as patients might have wanted to avoid hospitals due to fear of COVID-19 [12]. The aim of this study was to report the effects of the COVID-19 pandemic and its societal effects on the utilization of EMS due to trauma in Southern Finland, an area covering 1.3 million inhabitants.

## Methods

### Study design

We conducted a retrospective cohort study that included all EMS calls in the HUH area between 1 January and 31 July 2020 (study period). Data from EMS calls in 2018 and 2019 for the corresponding dates were used as reference (control period). The study plan and the conduction of the study were approved by the Institutional Review Board of Research and Education, Department of Emergency Medicine & Services, Helsinki University Hospital (HUS/247/2020). The aforementioned Board waived the need for ethics committee approval. Also, the Board waived the need for informed consent from patients. These waivers were based on Finnish research legislation, the Finnish Medical Research Act (488/1999). The study protocol was performed in accordance with the relevant guidelines. We reported the study in accordance with the Strengthening the Reporting of Observational studies in Epidemiology (STROBE) guideline [13].

### Study setting and population

Finland is a Nordic country with 5,500,000 inhabitants. Our study area, the HUH catchment area, is formed by the capital area of Helsinki and neighboring municipalities and has a total catchment population of 1,263,000 [14]. Although Helsinki is the largest urban center in Finland, the study area also contained less populated and rural areas.

### Emergency medical service

All emergency calls, regardless of type of emergency, are made to single number (112) and are handled by the national Emergency Response Center (ERC). The ERC operator handling the call follows a systematic questionnaire to assess the emergency and each mission is assigned a code that indicates the type of symptom, accident, injury, or violence. The ERC operator then dispatches the appropriate emergency services (i.e. rescue department, police, EMS, and other relevant agencies). Dispatched EMS units are assigned a triage class from A to D. Triage class A indicates a high-risk, life threatening situation with severe disturbance in vital signs or a high-risk injury. Class B indicates a situation in which there is a disturbance in vital signs that might progress to be life-threatening without prompt EMS interventions. Class A and B missions are responded to immediately with lights and sirens on. Class C indicates a situation where patient is stable and can wait for an ambulance. EMS responds within 30 min. Class D indicates a non-urgent situation. EMS responds within 120 min. Class C and D missions are responded to without lights and sirens on and by observing speed limits and regular traffic rules.

EMS in the HUH area is organized by the HUH and provided by three fire departments and two contracted private operators all operating under the same medical and operational guidelines. Ambulances are staffed by emergency medical technicians and paramedics. More advanced care in the area is provided by three on-duty medical supervisors and two prehospital physician units mainly responding to triage class A missions.

### The Finnish government response to the COVID-19 pandemic

On 12 March 2020, the Finnish government recommended canceling all mass gatherings and sport events and for all employees to work remotely from home if possible. The government declared a state of emergency on 16 March and implemented the Emergency Powers Act one day later. As a result, all gatherings of over 10 persons were banned, and schools and other educational institutions switched to distance learning with the exceptions of grades 1 to 3. Kindergartens were kept open only for children of persons employed in sectors critical to the functioning of society. All sport and culture venues were closed. Visits to institutional care facilities for the elderly were prohibited. Persons of over 70 years of age were instructed to self-quarantine. Many non-urgent health care services were suspended. Non-essential domestic and international travel was prohibited. Due to a regional spike in infections, Uusimaa, which is a region of 1,700,000 inhabitants and includes the capital Helsinki, was isolated from the rest of the country for the period from 28 March to 15 April. All restaurants, bars and nightclubs closed on 4 April at the latest, but many closed voluntarily before the deadline.

The relaxation of restrictions started with schools resuming classroom teaching on 14 May. On 1 June, many culture and sports venues and restaurants reopened and mass gatherings of up to 500 persons were permitted [15, 16].

### Study material

The HUH EMS uses an electronic health record (Merlot Medi®, CGI Finland Oy) and all EMS missions in the HUH area are registered in a single database. Patient data, including vital sign measurements and interventions are marked in the record. Alcohol intoxication is assessed by a breathalyzer test when it is suspected even slightly, and the blood alcohol level is marked in the record. In cases where patient refuses, or their condition does not allow for a breathalyzer test to be performed EMS personnel uses their clinical judgement. In such cases alcohol intoxication is marked as “positive” when it is obvious or if information concerning the patients’ alcohol use is available from other persons on scene. In addition to the patient data, mission data such as dispatch and transportation times, mission and triage codes, and possible non-conveyance data are recorded in the electronic health record.

We analyzed all EMS calls in the HUH area during the study and control periods. Calls were divided into the following three time periods: 1 January to 8 March “pre-lockdown”; 9 March to 31 May “lockdown”; and 1 June to 31 July “post-lockdown”. Trauma-related dispatch codes were identified; of these, all traffic injuries (i.e. road, rail, boat accidents), violence-related injuries (i.e. assaults, stabbings, shootings), and accidental injuries (i.e. falls, impacts, wounds, drownings, fire or chemical related injuries, electrocutions) were analyzed.

The “lockdown” period was determined to have started 1 week before the declaration of the state of emergency because of rising COVID-19 infection incidence, heavy press coverage on the subject, and unofficial calls for social isolation. The “lockdown” period was decided to have ended on 1 June, when restaurants, bars, and nightclubs were reopened and the restrictions on public gatherings were relaxed.

We analyzed separately the number of trauma-related EMS contacts for persons aged 0–16 years during school and kindergarten operation restrictions from 16 March to 14 May.

### Data analysis

The data are described using medians and interquartile ranges (IQR) and counts and percentages. Comparisons were performed for the number and percentage of weekly events using Mann-Whitney U test. Due to large dataset, *P*-values < 0.01 were considered significant. Analyses were performed using R version 3.6.3 and the ggplot2 package.

## Results

The total number of EMS calls during the study period was 70,705, of which 14,998 (21.2%) were related to trauma. During the control period, the mean corresponding numbers for 2018 to 2019 were 74,192 and 16,479.5 (22.2%). A total of 67,973 patients were met by the EMS during the study period; of these, 48.1% were male (median age 61.6 years; IQR 35.3–78.6). During the control period, the mean number of patients met was 71,096, of which 48.1% were male (median age 63.3 years; IQR 36.7–79.3). There were some missing data, sex and age being the most common missing variables with 4.1 and 3.9%, respectively, missing of all calls. Missingness of other variables was 0.3% at most. The missing observations were omitted from analyses.

The following results are reported as weekly median (IQR) and compared to the mean of medians from the corresponding weeks in 2018 to 2019.

### Pre-lockdown period comparison

There was no statistically significant change in the total number of EMS calls, trauma-related calls, trauma-related calls with injured patients intoxicated by alcohol, or trauma patients deceased on scene between the study and control periods (Table 1) The amount of triage class B calls increased by 21.4% (11.9–35.1%) and their proportion increased by 17.7% (13.3–35.2%).

**Table 1:** Pre-lockdown period

		Per week median (IQR)			
		Mean 2018–2019	2020	Change % (IQR)	P-value
**All EMS calls**	n	2405.0 (2386.0–2553.0)	2475.0 (2425.0–2507.0)	0.4 (− 1.1–1.0)	0,734
**Trauma-related calls**	n	517.0 (473.0–552.0)	518.0 (500.0–536.0)	− 0.1 (− 4.9 –11.5)	0,820
	% of all calls	21.5 (21.2–21.7)	21.4 (20.5–21.9)	1.5 (− 5.4– 5.0)	0,910
**Trauma-related calls –**	n	186.0 (181.0–192.5)	194.0 (186.0–210.0)	7.2 (2.8 –11.8)	0,039
**not conveyed**	% of all trauma-related calls	35.6 (35.1–37.5)	38.0 (37.7–39.6)	8.3 (1.2 –11.6)	0,027
**Traffic injuries**	n	59.5 (39.5–64.5)	65.0 (49.0–66.0)	10.9 (− 5.4 –21.5)	0,301
	% of all trauma-related calls	10.7 (8.3–12.2)	12.0 (9.6–13.2)	12.4 (− 6.0 – 26.2)	0,359
**Violence-related injuries**	n	33.0 (30.5–35.0)	44.0 (38.0–50.0)	31.3 (18.4 – 63.9)	0,055
	% of all trauma-related calls	6.8 (6.2–7.3)	8.5 (7.7–10.0)	19.5 (17.0 – 47.5)	0,039
**Accidental injuries**	n	429.0 (400.5–455.5)	418.0 (409.0–433.0)	− 1.9 ( − 8.2 – 3.4)	0,426
	% of all trauma-related calls	83.4 (80.9–84.7)	79.0 (77.5–81.4)	− 2.0 ( − 7.8 − 0.5)	0,020
**Trauma-related calls –**	n	124.0 (111.0–140.5)	129.0 (124.0–138.0)	5.8 (− 0.8 – 13.7)	0,164
**intoxicated by alcohol**	% of all trauma-related calls	25.1 (21.5–26.5)	24.2 (23.9–25.9)	11.2 (0.3 – 11.3)	0,129
**Intoxicated -**	n	2.5 (2.0–5.0)	4.0 (2.5–4.0)	0.0 (− 30.3 – 55.0)	1,000
**Traffic injuries**	% of all trauma-related calls	0.5 (0.4–1.0)	0.7 (0.5 - 0.8)	0.1 (− 26.8 – 47.3)	0,938
**Intoxicated -**	n	18.0 (16.0–19.5)	20.0 (17.0–28.0)	17.6 (− 10.5 – 40.0)	0,359
**Violence-related injuries**	% of all trauma-related calls	3.5 (3.3–4.0)	4.2 (3.2–5.4)	11.8 (− 21.4 – 47.2)	0,359
**Intoxicated -**	n	101.0 (89.0–120.0)	101.0 (99.0–120.0)	5.0 (1.2 – 15.3)	0,286
**Accidental injuries**	% of all trauma-related calls	21.5 (17.6–21.8)	20.2 (19.1–22.6)	6.4 (− 6.0 – 15.5)	0,426
**Triage class A**	n	14.0 (11.5–14.5)	16.0 (14.0–20.0)	9.1 (− 20.0 – 42.9)	0,406
	% of all triage classes	2.7 (2.5–2.8)	2.9 (2.7–3.6)	8.5 (− 18.9 – 46.3)	0,570
**Triage class B**	n	99.5 (97.0–109.0)	125.0 (122.0–130.0)	21.4 (11.9 – 35.1)	**0,008**
	% of all triage classes	19.3 (17.8–21.1)	24.4 (22.5–24.8)	17.7 (13.3 – 35.2)	**0,004**
**Triage class C**	n	268.0 (250.5–296.5)	252.0 (245.0–258.0)	− 10.6 (− 12.3 – 0.0)	0,141
	% of all triage classes	52.9 (50.8–53.0)	48.9 (46.6–49.8)	−7.5 ( − 10.2 − 4.8)	0,020
**Triage class D**	n	135.0 (129.0–139.0)	128.0 (120.0–131.0)	− 5.2 ( − 9.8 − 2.4)	0,155
	% of all triage classes	26.2 (25.6–26.2)	24.3 (23.4–26.2)	− 4.2 (− 12.6–1.9)	0,164

### Lockdown period comparison

**Fig. 1:**
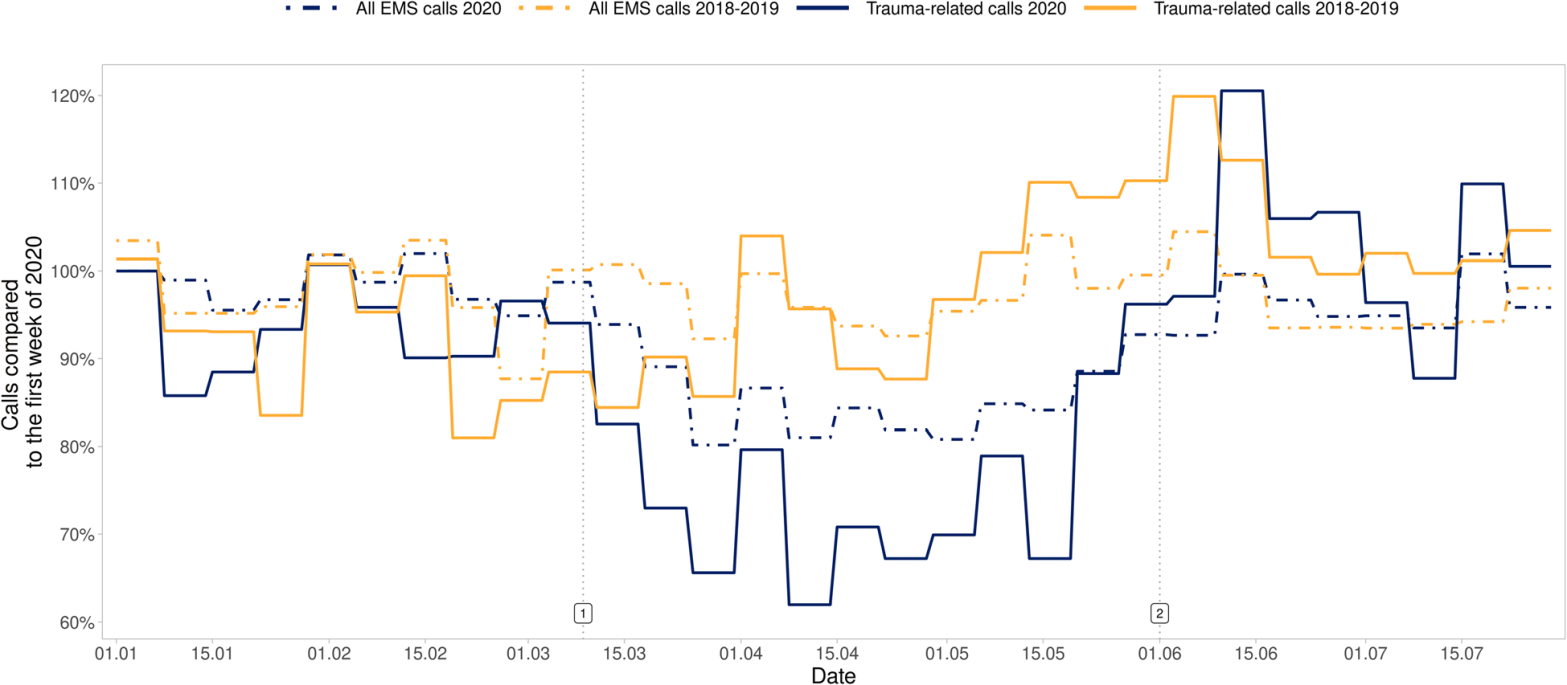
Percentage of weekly total EMS calls and trauma-related calls compared with the first week of 2020. Timeline: 1. 9 March, beginning of the “lockdown” period. 2. 31 May, end of the “lockdown” period

**Fig. 2:**
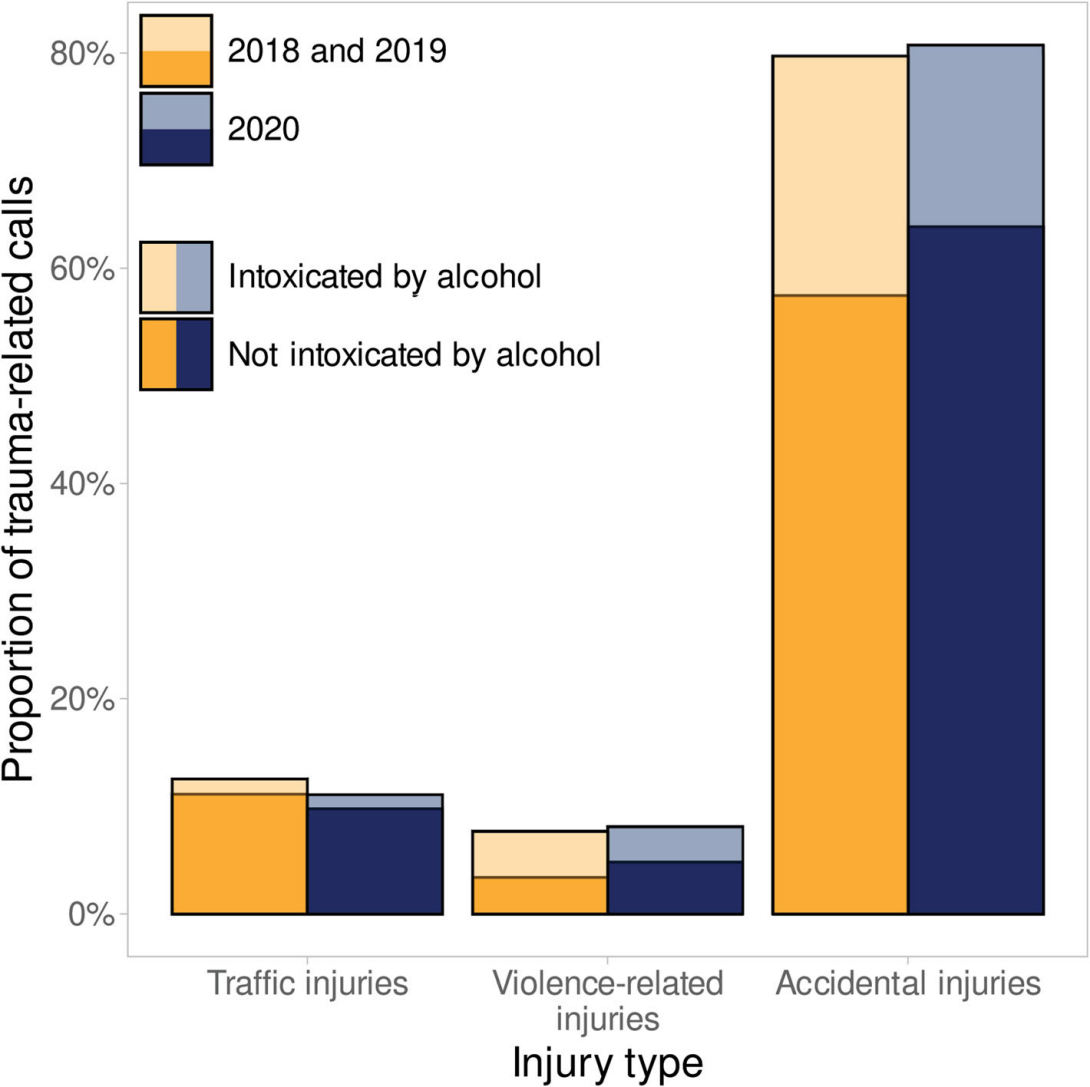
Proportion of trauma-related calls by injury type during lockdown period

**Fig. 3:**
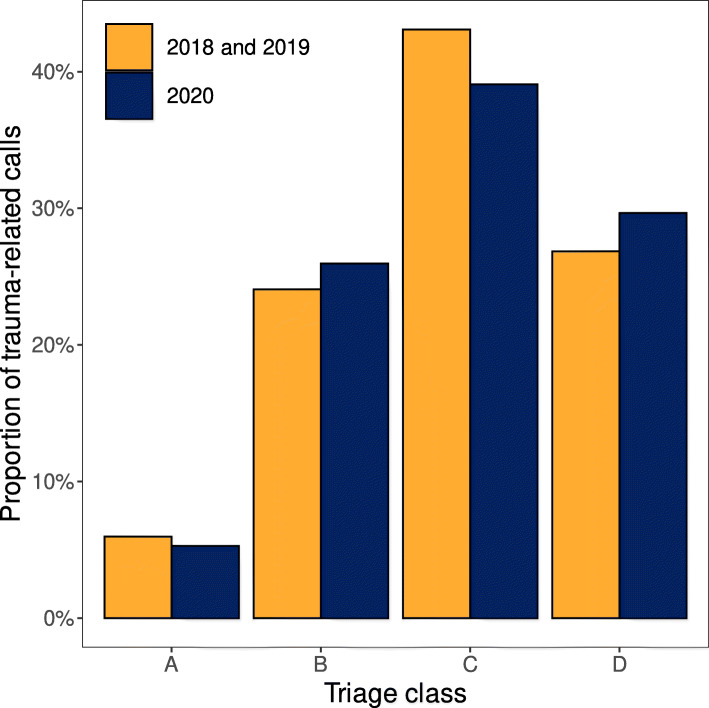
Proportion of trauma-related calls by dispatch triage class during lockdown period

**Fig. 4:**
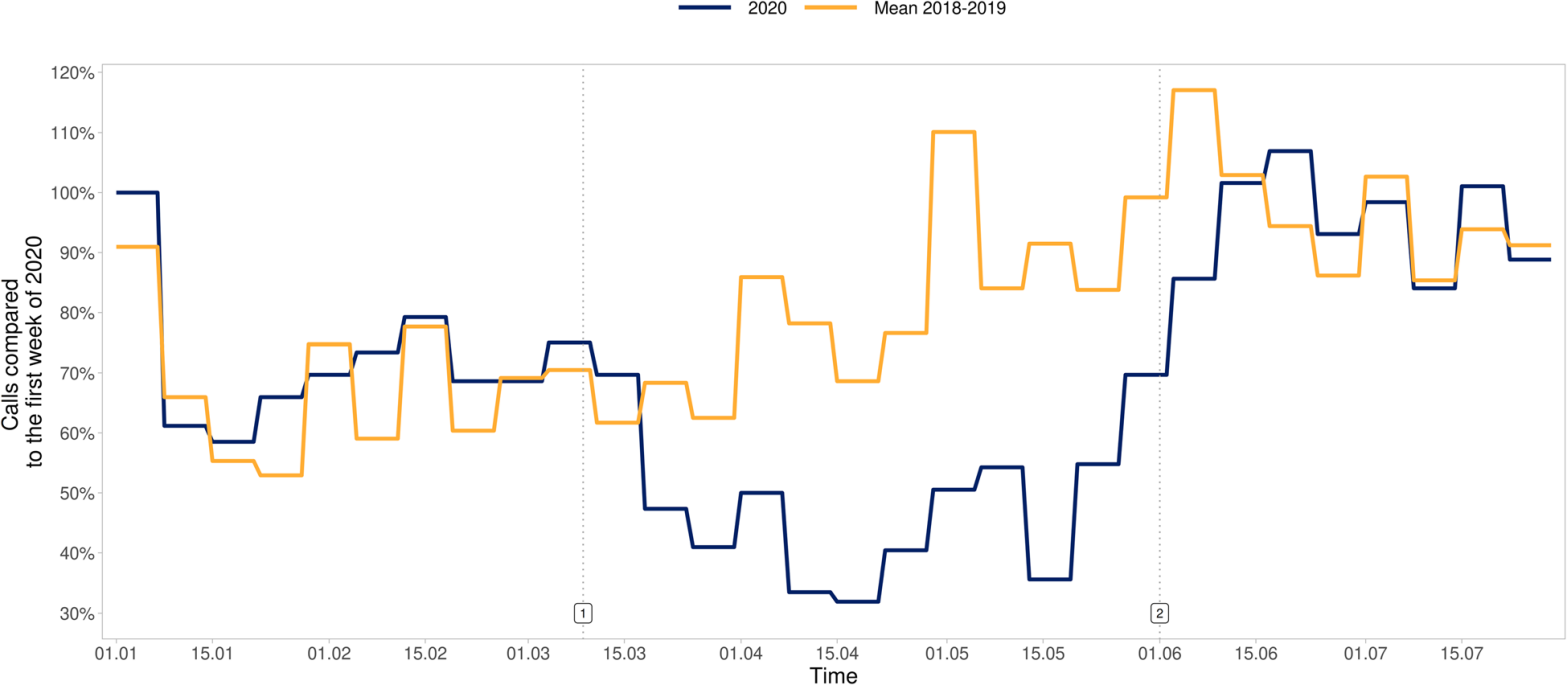
Percentage of weekly trauma-related EMS calls for patients intoxicated by alcohol. Timeline: 1. 9 March, beginning of the “lockdown” period. 2. 31 May, end of the “lockdown” period

The total amount of EMS calls decreased by 12.2% (− 14.2% to − 9.8%), while trauma-related calls decreased by 23.3% (− 25.6% to − 19.7%) and their proportion of all calls by 11.9% (− 14.0 to − 11.0%) (Fig. 1), with no statistically significant change in the distribution of trauma categories or triage classes (Table 2, Figs. 2 and 3). The number of calls with injured patients intoxicated by alcohol decreased by 41.8% (− 53.8% to − 34.5%) (Fig. 4) and their proportion of all trauma-related calls decreased by 24.2% (− 34.9 to − 15.6%). The number of calls with patient being intoxicated in violence-related injury subgroup decreased by 51.6% (− 57.0% - -32.5%). In subgroup of accidental injury, the number of calls with intoxicated patient decreased by 42.0% (− 56.0% - -32.8%) and their proportion of all accidental injuries decreased by 24.6% (− 33.8 - -17.3%). There was no statistically significant change in the non-conveyance rate or number of patients deceased on scene.

**Table 2:** Lockdown period

		Per week median (IQR)			
		Mean 2018–2019	2020	Change % (IQR)	P-value
**All EMS calls**	n	2423.0 (2370.8–2485.2)	2115.0 (2042.0–2196.0)	−12.2 (− 14.2 – 9.8)	**0,001**
**Trauma-related calls**	n	531.0 (489.8–571.8)	393.0 (373.0–440.0)	−23.3 (− 25.6 – 19.7)	**0,004**
	% of all calls	22.1 (20.8–23.3)	18.6 (18.1–19.9)	− 11.9 (− 14.0 – 11.0)	**0,002**
**Trauma-related calls –**	n	199.0 (178.2–218.5)	163.0 (146.0–172.0)	− 12.3 (− 26.3 – 5.2)	**0,002**
**not conveyed**	% of all trauma-related calls	37.3 (36.4–37.8)	39.5 (37.9–42.8)	7.4 (−1.5–17.1)	0,123
**Traffic injuries**	n	58.0 (52.5–73.8)	40.0 (38.0–44.0)	−32.1 (− 39.3 – 24.0)	**0,002**
	% of all trauma-related calls	10.9 (10.0–14.0)	10.4 (9.4–11.2)	−8.7 (− 20.9 – 2.1)	0,054
**Violence-related injuries**	n	42.5 (34.8–48.2)	34.0 (26.5–36.5)	− 29.4 (− 40.6 – 2.0)	0,068
	% of all trauma-related calls	7.6 (7.2–8.5)	8.2 (7.3–9.0)	− 5.2 (− 13.2 – 26.3)	0,577
**Accidental injuries**	n	429.5 (401.5–438.8)	323.0 (307.0–358.0)	−22.4 (− 25.8 – 18.8)	**0,001**
	% of all trauma-related calls	80.6 (77.8–82.7)	82.0 (80.7–82.1)	1.9 (− 1.8 – 4.5)	0,365
**Trauma-related calls –**	n	147.0 (128.8–159.8)	89.0 (71.5–98.5)	− 41.8 (− 53.8 – 34.5)	**0,002**
**intoxicated by alcohol**	% of all trauma-related calls	27.6 (26.0–28.1)	21.2 (19.3–22.6)	−24.2 (− 34.9 – 15.6)	**0,005**
**Intoxicated -**	n	6.5 (4.0–10.0)	4.0 (3.0–5.5)	− 50.0 (− 55.6 – 7.9)	0,066
**Traffic injuries**	% of all trauma-related calls	1.3 (0.8–1.9)	1.1 (0.7–1.5)	− 31.2 (− 41.2 – 37.1)	0,413
**Intoxicated -**	n	22.0 (19.2–28.0)	13.0 (12.0–14.5)	−51.6 (− 57.0 – 32.5)	**0,007**
**Violence-related injuries**	% of all trauma-related calls	4.1 (4.0–4.8)	3.3 (2.7–3.6)	−31.4 (− 40.3 – 13.4)	0,054
**Intoxicated -**	n	116.0 (104.2–125.2)	70.0 (55.5–78.5)	−42.0 (− 56.0 – 32.8)	**0,005**
**Accidental injuries**	% of all trauma-related calls	21.2 (20.9–23.0)	17.2 (15.7–18.2)	−24.6 (− 33.8 – 17.3)	**0,002**
**Triage class A**	n	16.0 (13.2–20.0)	13.0 (11.5–15.0)	− 11.1 (− 34.3 – 5.6)	0,041
	% of all triage classes	3.2 (2.6–4.1)	3.3 (2.8–3.8)	11.1 (− 13.3 – 20.1)	0,638
**Triage class B**	n	100.0 (92.8–129.5)	85.0 (77.0–93.0)	− 16.7 (− 36.8 – 7.2)	0,016
	% of all triage classes	19.4 (18.6–21.7)	21.2 (19.2–23.1)	13.9 (− 11.2 – 21.1)	0,700
**Triage class C**	n	277.5 (261.5–293.8)	188.0 (180.0–225.5)	− 30.5 (− 35.4 – 17.5)	**0,001**
	% of all triage classes	53.4 (50.3–53.7)	49.9 (46.6–51.7)	−8.3 (− 11.6 – 1.9)	0,147
**Triage class D**	n	130.0 (117.5–131.8)	105.0 (96.0–114.5)	− 10.9 (− 25.9 – 8.3)	**0,004**
	% of all triage classes	23.0 (22.2–25.5)	26.0 (24.0–26.7)	14.5 (0.7 − 18.2)	0,102

### Post-lockdown period comparison

There were no statistically significant changes in any studied parameters (Table 3).

**Table 3:** Post-lockdown period

		Per week median (IQR)			
		Mean 2018–2019	2020	Change % (IQR)	P-value
**All EMS calls**	n	2358.2 (2345.5–2467.2)	2391.0 (2368.8–2442.5)	0.7 (− 0.9 – 2.0)	0,742
**Trauma-related calls**	n	564.8 (559.5–591.6)	573.0 (538.0–596.5)	0.2 (− 7.1 – 7.0)	0,945
	% of all calls (IQR)	23.9 (23.6–24.4)	23.5 (23.0–24.4)	−0.6 (− 7.2 – 2.3)	0,547
**Trauma-related calls – not conveyed**	n	210.8 (205.2–224.5)	208.5 (201.0–225.2)	−1.5 (− 4.8 – 3.7)	0,844
	% of all trauma-related calls	36.8 (36.1–37.2)	37.5 (36.5–37.9)	0.6 (− 3.1 – 4.8)	0,641
**Traffic injuries**	n	87.5 (81.1–91.8)	75.0 (69.5–102.5)	0.5 (− 17.1 – 15.5)	0,833
	% of all trauma-related calls	15.1 (14.4–16.0)	13.2 (12.6–16.9)	1.0 (− 16.5 – 13.7)	0,641
**Violence-related injuries**	n	50.0 (46.0–52.8)	50.0 (48.0–55.5)	9.4 (− 2.4 – 15.2)	0,641
	% of all trauma-related calls	8.5 (8.0–9.5)	8.9 (8.5–9.6)	8.8 (− 1.5 – 12.8)	0,461
**Accidental injuries**	n	438.2 (426.1–460.9)	434.5 (418.5–454.2)	−1.4 (− 9.5 – 3.4)	0,641
	% of all trauma-related calls	76.5 (75.4–77.4)	77.1 (75.0–78.3)	0.6 (− 3.9 – 2.2)	1,000
**Trauma-related calls –**	n	177.0 (169.1–193.1)	180.0 (165.5–190.2)	−1.4 (− 3.0 – 7.7)	1,000
**intoxicated by alcohol**	% of all trauma-related calls	31.2 (29.3–31.9)	30.5 (29.8–32.9)	1.2 (− 2.7–3.5)	0,641
**Intoxicated -**	n	15.8 (13.8–17.5)	19.0 (12.5–24.2)	36.2 (1.4 – 59.6)	0,726
**Traffic injuries**	% of all trauma-related calls	2.7 (2.3–3.0)	3.3 (2.5–3.8)	39.3 (5.3 – 49.4)	0,547
**Intoxicated -**	n	28.2 (23.4–31.4)	22.0 (21.8–23.2)	−16.8 (− 28.0 – 2.7)	0,055
**Violence-related injuries**	% of all trauma-related calls	4.8 (4.1–5.5)	3.9 (3.7–4.3)	− 21.4 (− 24.7 – 4.8)	0,055
**Intoxicated -**	n	135.5 (126.2–149.1)	136.5 (129.0–146.0)	0.5 (− 5.1 – 7.4)	0,889
**Accidental injuries**	% of all trauma-related calls	23.7 (22.1–24.6)	24.0 (23.1–25.7)	4.1 (− 0.6 – 7.4)	0,383
**Triage class A**	n	22.2 (17.9–28.2)	22.5 (19.8–27.0)	14.1 (− 10.4 – 23.9)	0,726
	% of all triage classes	3.9 (3.2–4.7)	4.1 (3.4–5.0)	7.0 (− 11.2 – 23.2)	0,547
**Triage class B**	n	117.8 (114.5–127.0)	129.5 (108.5–138.0)	3.8 (− 9.4 – 13.7)	0,674
	% of all triage classes	20.7 (20.2–21.2)	21.1 (20.6–22.3)	2.9 (− 2.6 – 7.7)	0,547
**Triage class C**	n	305.5 (299.0–313.0)	317.0 (292.2–331.8)	4.6 (− 4.6 – 12.3)	0,547
	% of all triage classes	53.3 (52.4–54.7)	55.7 (55.0–56.0)	4.4 (− 1.3 – 8.2)	0,148
**Triage class D**	n	126.2 (123.6–128.6)	115.5 (95.8–117.5)	−10.3 (− 24.5 – 4.3)	0,023
	% of all triage classes	22.3 (20.3–22.8)	18.7 (17.1–20.0)	−12.5 (− 26.1 – 5.8)	0,055

### School and kindergarten operation restrictions

The number of trauma-related contacts for patients aged 0 to 16 years decreased by 18.9% (− 25.5% to − 7.4%) during school and kindergarten opening restrictions.

## Discussion

### Key results

We observed a remarkable reduction (23%) in trauma-related EMS activations and a minor reduction (12%) in all EMS calls during the lockdown phase of the COVID-19 pandemic.

The reduction was even more significant for trauma-related calls with the patients intoxicated by alcohol; the weekly median decreased from 147 to 89 calls. The numbers of all aforementioned call types returned to the previous years’ levels in the 2 months after relaxation of lockdown measures. During the lockdown, there was no significant change in the distribution of different trauma types or triage classes. In the post-lockdown period, triage class D calls decreased slightly with no statistically significant increase in the other triage classes. Trauma calls for patients aged 0 to 16 during the restrictions for kindergarten and school operations were significantly reduced (− 18.9%).

We included the pre-lockdown period to account for possible changes in EMS calls in 2020 not related to the COVID-19 pandemic. Compared with previous years, during the pre-lockdown period there were no statistically significant changes in the number of total calls, trauma-related calls, or calls for patients intoxicated by alcohol. There was a higher proportion of triage class B calls. This could be explained by the new automated ERC dispatch system implemented in May 2019 that affected triage classification.

### Relation of results to other studies

There are only a few studies [1–4] that have described the incidence of prehospital trauma during the pandemic, and all showed a similar trend in reduction of cases during the first pandemic wave. In the study by Lerner et al. that documented EMS activations in the United States, the number of EMS activations between week 10 and week 16 decreased by 26% and the number of EMS activations with suspected injury decreased from 18 to 15% between weeks 10 and 13. In Israel, Jaffe et al. found a 24% reduction in injury-related EMS calls between 03 March and 15 April 15, 2020, compared to the time period of 01 December 2019 and March 2, 2020.

To our knowledge there have been no previous reports on the effect of COVID-19 lockdown on the incidence of injury while intoxicated in the prehospital setting. A study by Kreis et al [7] reported a 30% reduction in overall cases and a 70% reduction of alcohol intoxication / abuse as a mechanism of injury at a Level 1 trauma center emergency department in Germany during 16 March to 19 April 2020 compared to the previous year. In a study by Navsaria et al [17] from South Africa, a significant reduction of violent and traffic injury during lockdown was in part attributed to the ban on alcohol sales during the lockdown. These findings might suggest that alcohol is a significant confounder in injury and changes affecting its availability and societal circumstances of its usage influence the incidence of injury.

### Relevance of the study results

The pandemic provided a unique social setting with cultural institutions and nightlife shutting down for almost 2 months. During this time, the number and proportion of alcohol-related trauma calls decreased dramatically. Alcohol is a significant contributor to injury-related mortality and morbidity [18] and exerts a social and monetary cost on societies. In normal circumstances, in Finland and elsewhere, a considerable portion of patients presenting to EDs due to trauma are intoxicated [19, 20]. To cope in part with the increased workload brought on by intoxicated patients, EMS and EDs are more heavily resourced during nights and weekends as these are typically times of the highest demand. A decrease in this patient group frees healthcare resources to be allocated to treat other patient groups (i.e. COVID-19 patients). Decrease in the number of patients with injuries reduces the need for surgery and frees anesthetists and anesthetic nurses to treat the increased number of critically ill patients during the peaks of the pandemic. Also, decreased demand for workforce during inconvenient work hours allows for more resting time to medical professionals burdened by challenges brought on by the pandemic. After the end of the lockdown, the number of overall and trauma-related EMS calls returned to previous years’ levels. This suggests that the changes in the demand can and should be anticipated and that mechanisms for resource allocation should be flexible.

The distribution of trauma types was unchanged. While traffic-related injuries decreased expectedly, violence-related injuries and accidental injuries decreased as well. The reduction in violence-related injuries could be attributed to closure of nightlife, while the general reduction in mobility of individuals, the cancellation of sport events and practices, and the reduction of accidents among children during school closure decreased the number of accidental injuries. The non-conveyance rate remained unchanged during the lockdown, suggesting that attitudes of EMS personnel or patients’ fear of contracting COVID-19 in EDs did not affect transportation decisions during the lockdown.

The population in the catchment area of the study covers almost a quarter of the total population of Finland and includes not only the capital city but also residential and semi-rural areas. All emergency calls are made to the ERC and the EMS organized by HUH is the only EMS service in the area. All calls and EMS contacts are automatically registered in the database at the time of EMS dispatch and mission data is updated on-line until the end of each EMS mission. Thus, our data provide a reliable and comprehensive view on the subject.

### Limitations and future studies

This was a retrospective registry analysis. We reviewed trauma types only in a general manner and did not account for the site or the time of day of EMS calls. These data could provide more specific insights into changes in trauma incidence during the pandemic. As only EMS electronic health record data was used for this study, no data on prognosis or injury severity was available. A recent study by Riuttanen et al [21] showed that the incidence of severe injury (New Injury Severity Score > 15) did not significantly decrease during lockdown in Finland. During pandemic there have been concerns about increased mental health issues during lockdown conditions. Our data did not permit to differentiate self-inflicted trauma and thus overall reduction in trauma-related calls might not reflect changes in self-inflicted trauma incidence during the lockdown.

## Conclusions

The societal restrictions imposed by the Finnish government to curb the spread of COVID-19 had a significant effect on the number of EMS calls related to trauma in the capital area. The number of injured patients intoxicated by alcohol decreased significantly and the decrease was temporally related to the lockdown which included the closure of bars and nightclubs.

## Data Availability

The data used in the study were not publicly available. The permission to use the data was included in the approval of the conduction of the study granted by Institutional Review Board of Research and Education, Department of Emergency Medicine & Services, Helsinki University Hospital. The data analyzed during the current study are available from the corresponding author on reasonable request.

## Abbreviations

EMS: Emergency medical service
HUH: Helsinki University Hospital
ED: Emergency department
ERC: Emergency response center
